# A set of synthetic versatile genetic control elements for the efficient expression of genes in Actinobacteria

**DOI:** 10.1038/s41598-017-18846-1

**Published:** 2018-01-11

**Authors:** Lilya Horbal, Theresa Siegl, Andriy Luzhetskyy

**Affiliations:** 1 Department Microbial Natural Products, Actinobacteria Metabolic Engineering Group, Saarland University, Saarbrücken, Germany; 2 Helmholtz-Institute for Pharmaceutical Research Saarland (HIPS) Helmholtz Center for Infectious Research (HZI), Campus C2.3, 66123 Saarbrücken, Germany

## Abstract

The design and engineering of secondary metabolite gene clusters that are characterized by complicated genetic organization, require the development of collections of well-characterized genetic control elements that can be reused reliably. Although a few intrinsic terminators and RBSs are used routinely, their translation and termination efficiencies have not been systematically studied in Actinobacteria. Here, we analyzed the influence of the regions surrounding RBSs on gene expression in these bacteria. We demonstrated that inappropriate RBSs can reduce the expression efficiency of a gene to zero. We developed a genetic device – an *in vivo* RBS-selector – that allows selection of an optimal RBS for any gene of interest, enabling rational control of the protein expression level. In addition, a genetic tool that provides the opportunity for measurement of termination efficiency was developed. Using this tool, we found strong terminators that lead to a 17–100-fold reduction in downstream expression and are characterized by sufficient sequence diversity to reduce homologous recombination when used with other elements. For the first time, a C-terminal degradation tag was employed for the control of protein stability in *Streptomyces*. Finally, we describe a collection of regulatory elements that can be used to control metabolic pathways in Actinobacteria.

## Introduction

For decades, metabolic engineers and synthetic biologists have been attempting to develop principles for biological engineering from the “ground up” to allow the rational construction of complex circuits and systems with balanced expression of genes using suitable building blocks. Such synthetic circuits and systems should exhibit temporal and spatial control and should be orthogonal, not restricted to the regulatory machinery of a host cell. Genetic information in the cell is transferred from DNA to RNA and from RNA to protein via transcription and translation, respectively. Many structural elements, such as promoters, ribosomal binding sites (RBSs), terminators, and 5′- and 3′-untranslated regions (UTRs), influence the efficiency of these processes. In addition, a myriad of regulatory proteins and small non-coding RNAs (e.g., riboswitches, ribozymes) govern the expression of a gene. Thus, a precise understanding of the regulation of gene expression at the above mentioned levels and the interaction of regulatory genetic elements is needed. Towards this goal, libraries of natural and synthetic controlling elements that influence gene expression on different levels in *Escherichia coli* and *Saccharomyces cerevisiae* have been studied, and numerous synthetic control modules are being developed^1–5^. However, there is a deficiency of the building blocks described above for less-studied but highly industrially important bacteria, such as Actinobacteria.

With the advent of next-generation sequencing techniques, it became obvious that the biosynthetic potential of Actinobacteria has been underestimated, because their genomes contain a hidden wealth of silent secondary metabolite gene clusters^6–8^. The problem of the existence of dormant or unexpressed gene clusters is mainly related to the intricate and tight regulatory networks that precisely orchestrate metabolite production in bacteria and respond to various environmental and intracellular signals^9,10^. One of the strategies for overcoming this obstacle is the decoupling of metabolite biosynthesis from the regulatory networks that exist in the cell by placing genes in a cluster under the control of constitutive or orthogonal inducible promoters. A lot of effort has been made to develop engineering elements that govern the expression of genes at the transcriptional level^11–19^. However, controlling elements, such as RBSs, terminators and degradation tags, that influence the remaining aspects of the process and may be used to balance expression, and thus control output, have remained overlooked in Actinobacteria. The complexity of secondary metabolite gene clusters^20,21^ results from the existence of bottlenecks that require the expression of multiple gene products from individually controlled gene sets; hence, there is demand for libraries of promoters, RBSs and terminators. In addition, sequences with minimal identity are needed to avoid homologous recombination with synthetic operons and consequent genetic instability. One additional hurdle that should be considered during the design of metabolite gene clusters is the genetic context, which may influence the activity of elements such as promoters and RBSs^22,23^. Therefore, there is an urgent need for diverse regulatory elements, such as promoters, RBSs, terminators, and genetic tools that will allow researchers to measure, characterize and select appropriate controlling elements for a gene of interest in Actinobacteria.

Herein, we report the development of a genetic tool – an *in vivo* RBS-selector – for the selection of an optimal synthetic RBS for any gene of interest, enabling rational control of the protein expression level. The designed methodology allows a single experiment to test the activity of a huge number of randomly synthesized RBSs and choose the best RBS with the required activity that is suitable for a certain gene. Furthermore, this tool provides the possibility of selecting an optimal RBS for a gene of interest in combination with any promoter, due to the fact that the genetic context of the latter element may influence the activity of the former. Using this tool, we generated a library of synthetic RBSs for the *gusA* gene^24^, resulting in a number of reporters with different expression strengths and, consequently, a flexible dynamic range. In addition, a genetic tool for the reliable measurement of termination efficiency and a library of intrinsic terminators that play an important role in transcriptional regulation were developed for *Streptomyces*. For the first time, an 11-amino acids C-terminal degradation tag was used for the regulation of gene expression at a posttranslational level in *Streptomyces*, and several variants of the unstable GusA reporter were constructed. In conclusion, the combination of the control elements described above provides the possibility of spatial, temporal and quantitative regulation of gene expression at transcriptional and/or translational levels, thereby facilitating the ability to overcome bottlenecks and obtain greater amounts of compounds as well as toxic and non-detrimental proteins. The described features make these tools very promising for metabolic engineering and biotechnology of *Streptomyces* and other Actinobacteria.

## Results

### The nucleotides surrounding the Shine-Dalgarno domain dictate the efficiency of translation initiation

Engineering and development of systems that control the expression of genes are in most cases focused on the transcriptional level^13,14,16,17^. However, in *E*. *coli* and some other bacteria, translation initiation is the rate-limiting step in the translation process and can strongly influence protein synthesis^25–27^. Changes in the sequence, shape and structure of the translation initiation region (TIR) alter mRNA folding, which in turn affects the thermodynamic energy barrier that ribosomes must cope with to form a stable initiation complex and initiate translation^1,26,28,29^. Therefore, there is a need to engineer efficient genetic elements that control the expression of genes on posttranscriptional or translational levels, as these are additional critical steps for the production of proteins.

**Figure 1:**
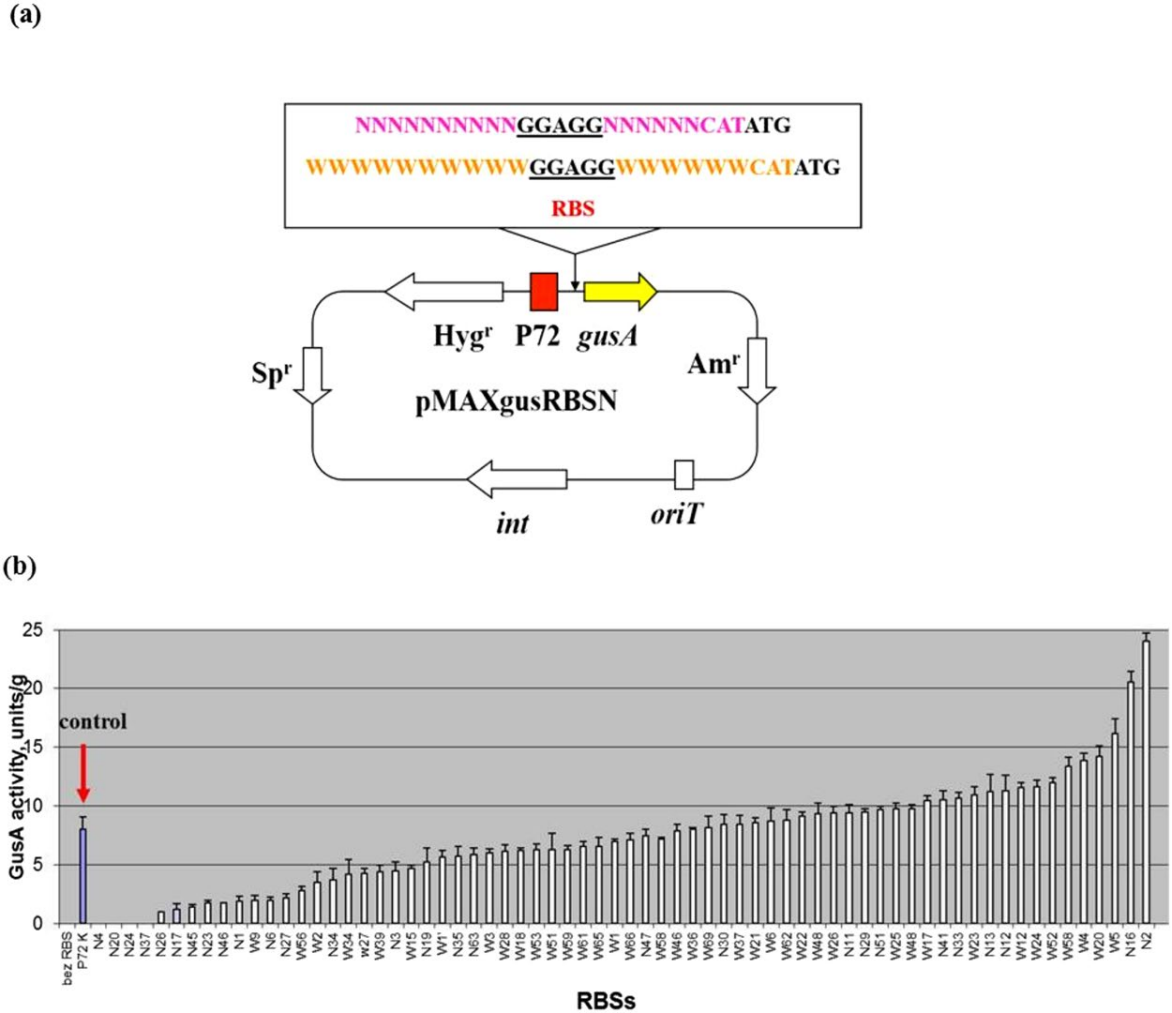
Scheme of random RBSs construction and estimation of their activity. (**a**) Schematic representation of the RBS containing plasmids used in the study. *gusA*, reporter gene; P72, promoter; Hyg^r^, Sp^r^ and Am^r^, hygromycin, spectinomycin and apramycin resistance genes, respectively; *int*, integrase gene; N – A, T, G or C; W – A or T. (**b**) Glucuronidase activity in cell lysates from recombinant *S*. *lividans* strains containing *gusA* under the control of the P72 promoter fused to different synthetic RBSs. The strains were grown in TSB medium for 2 days. Error bars indicate the standard deviations of three independent experiments.

**Figure 2:**
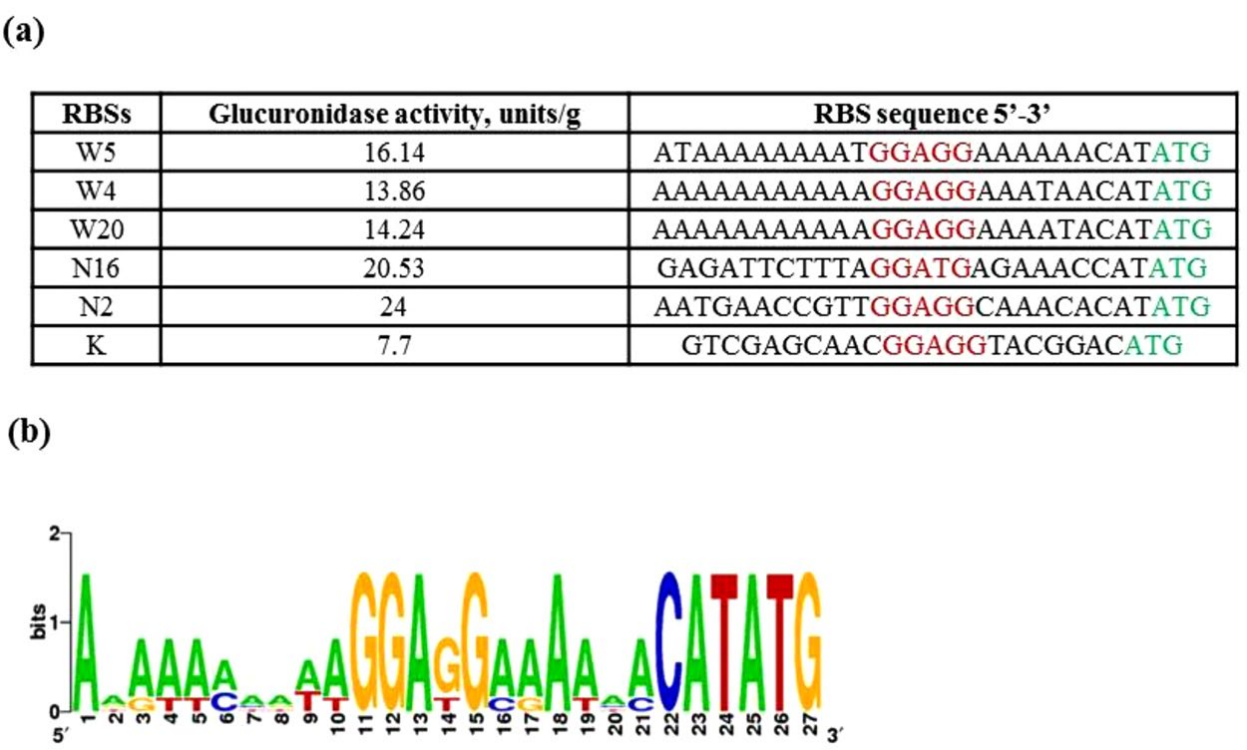
The strongest RBSs obtained in this work. (**a**) Sequences of the strongest RBSs and their activity. (**b**) WebLogo analysis of the strongest RBSs. WebLogo was generated using WebLogo 3 (http://weblogo.threeplusone.com/).

There are currently no available data on the efficiency of translation initiation in *Streptomyces*. Therefore, we decided to estimate how changes in the sequence of 5′ untranslated regions (UTRs) can influence translation in these bacteria and in *S*. *lividans* TK24 (Table 1) in particular. A library of synthetic RBSs for the *gusA* reporter gene was constructed (Table S1). For this purpose, the weakest known constitutive promoter, P72, from our library of semi-synthetic promoters^16^ was fused with the randomly synthesized RBSs and the *gusA* reporter gene to allow validation of the translation efficiency based on glucuronidase activity. The generated synthetic RBSs contain a consensus Shine-Dalgarno domain, “GGAGG”, which was fixed to a length of 5 bp because it has been shown that expression levels in *E*. *coli* decrease when the sequence is expanded to more than six nucleotides^30^. The nucleotides surrounding the SD region, up to 10 bp upstream and 6 bp downstream, were randomly synthesized (Fig. 1a). Two types of degenerate primers were used in this work (Fig. 1a). One type of primer contained N-type random nucleotides, where N is any base (A, T, G or C). The other type of primer contained W-type random nucleotides, where W is either an A or T base. Two types of primers were used, since it was shown that AT-rich RBSs provide better level of translation in *E*. *coli*^31,32^. The weakest P72 promoter was employed so that even very small changes in translation could be observed. The use of degenerate XbaRBSForw and NdeNRBSRev or XbaRBSForw and NdeWRBSRev primer pairs (Table 2), respectively, and an amplified hygromycin resistance gene fused to outward oriented P72 promoter facilitated the rapid and easy cloning of a variety of different synthetic RBSs upstream of the *gusA* gene (for details see Materials and Methods). The obtained library of synthetic RBSs was sequenced, and 70 different variants were chosen for further investigation. An additional plasmid without an RBS between the P72 promoter and the *gusA* gene was constructed. In this plasmid, the reporter gene was directly fused with the promoter. All 70 plasmids carrying different RBS regions were transferred to the *Streptomyces lividans* TK24 strain via tri-parental conjugation with *E*. *coli*. Exconjugants were selected on the basis of their resistance to apramycin and hygromycin. Consequently, 70 recombinant strains that harbored different RBSs were obtained. All of these strains were grown for 2 days in liquid TSB medium and then subjected to quantitative measurement of glucuronidase activity for the indirect analysis of RBS activity. The results of the analysis are depicted in Fig. 1b. As shown in the figure, we obtained couple RBSs (W5, W4, W20, N2 and N16) that were approximately 2–3 times stronger than the control, and there were also several that were much weaker. Four of the 70 RBSs, namely N4, N20, N24 and N37, could severely impair translation efficiency by decreasing the translation level to 0. The sequences of the strongest RBSs (Fig. 2) that we succeeded in creating exhibited greater AT richness, which is in accord with the previously reported data from *E*. *coli*^31,32^. Summing up, the sequences of the nucleotides located up- and downstream from the SD domain can clearly strongly affect translation efficiency.

**Table 1:** Strains and plasmids used in this study.

**Bacterial strains and plasmids**	**Description**	**Source or reference**
*S*. *albus* J1074	Isoleucine and valine auxotrophic derivative of *S*. *albus* G (DSM 40313) lacking *Sal*I-restriction activity	Salas J., Oviedo, Spain
*S*. *lividans* TK24	Derivative of *S*. *lividans* TK21 that contains mutation in the *rpsL* gene and is resistant to spectinomycin	^41^
*E*. *coli* DH5α	Routine cloning	MBI Fermentas
*E*. *coli* ET12567 (pUZ8002)	Conjugative transfer of DNA	^41^
pGUS	Promoter probe vector containing promoterless *gusA*	^24^
pUC19	Ap^r^, general cloning vector	MBI Fermentas
pSET152	Am^r^; φC31-based integrative vector	^41^
pGUSHL4aadA	pTESa-based vector for translational fusion with *gusA*	^24^
pGUSbezRBS	Derivative of pGUS containing the *gusA* gene without promoter and RBS	This work
pGUSP72bezRBS	Derivative of pGUS containing the *gusA* gene with the P72 promoter but without RBS	This work
pGUSRBSProg1	Derivative of pGUSbezRBS containing synthetic RBS generated using RBS calculator	This work
pGUSNRBS-8-3	Series of pGUSbezRBS derivatives containing NRBS-2 that differ in the size of the insert separating the SD from the ATG	This work
pSETP82Ap	Derivative of pSET152 containing ampicillin resistance gene fused with the P82 promoter	This work
pGUSNRBSEGFP	Derivative of pGUSHL4aadA containing randomly generated RBSs fused with first 60 bp of the *egfp* gene and *gusA* gene	This work
pEGFPN-9	Derivative of pGUS containing the *egfp* gene fused with the P82 promoter and RBSN-9	This work
pEGFPN-131	Derivative of pGUS containing the *egfp* gene fused with the P82 promoter and RBSN-131	This work
pGUSSPL21Termin	Derivative of pGUS containing six different terminators inserted downstream of the P21 promoter	This work
pGCymRP21	Derivative of pGUS containing the gusA gene under the control of the P21-cmt promoter	^18^
pGCymRP21-LVA	Derivative of pGCymRP21 containing *gusA* gne fused to the C-terminal degradation tag	This work
pGCymRP21-ASV	Derivative of pGCymRP21 containing *gusA* gne fused to the C-terminal degradation tag	This work
pGCymRP21-AAV	Derivative of pGCymRP21 containing *gusA* gne fused to the C-terminal degradation tag	This work

**Table 2:** Primers used in this study.

**Primers**	**Sequence 5′-3′**	**Purpose**
XbagusAForw	AAAATCTAGATACGCATATGCTGCGGCCCGTCGAAACC	Cloning the *gusA* gene without RBS and promoter
EVgusARev	AAAAGATATCTGCTTCCCGCCCTGCTGCGG	
XbaRBSForw	AAAAGATATCGAAATCACTCCCAATTAATCTAG	Cloning randomly synthesized RBSs upstream of the *gusA* gene
NdeNRBSRev	AAAACATATGNNNNNNCCTCCNNNNNNNNNNTTTCTCATCCTAAAGAATCTCTC	
NdeWRBSRev	AAAACATATGWWWWWWCCTCCWWWWWWWWWWTTTCTCATCCTAAAGAATCTCTC	
NdeP72bezRBS	AAAACATATGTTTCTCATCCTAAAGAATCTCTC	Cloning the *gusA* gene with the P72 promoter but without RBS
RBSProg1Rev	AAAACATATGAATGAACCTCCTTCTTTCTTTTTCTCATCCTAAAGAATCTCTC	Cloning the *gusA* gene fused to *in silico* designed RBS
NdeRBS8Nrev	AAAACATATGTGTTTCCTCCAACGGTTCATTTTCTCATCCTAAAGAATCTCTC	Cloning *gusA* fused to synthetic RBSs
NdeRBS7Nrev	AAAACATATGTGTTCCTCCAACGGTTCATTTTCTCATCCTAAAGAATCTCTC	
NdeRBS6Nrev	AAAACATATGTGTCCTCCAACGGTTCATTTTCTCATCCTAAAGAATCTCTC	
NdeRBS5Nrev	AAAACATATGTGCCTCCAACGGTTCATTTTCTCATCCTAAAGAATCTCTC	
NdeRBS3NRev	AAAACATATGCCTCCAACGGTTCATTTTCTCATCCTAAAGAATCTCTC	
ApForw	TTTTTCTAGTTATATGAGTAAACTTGGTCT	Fusion the Ap^r^ gene with the P82 promoter
ApP82Rev	GCTACAATCCTACTTGAAGAATCCTAATTTTAGCCTCAGGAGACGAAAGGGCCTCGTGATA	
EGFPNRBSRev	TTTTGATATCGAACAGCTCCTCGCCCTTGCTGGATCGGGATCCTTTTTCGAACTGCGGGTGGCTCCACATNNNNNNCCTCCNNNNNNNNNGCTACAATCCTACTTGAAGAATC	Cloning randomly synthesized RBSs upstream of the *egfp* gene
EGFPNRBS-9For	TTTTTCTAGATCCTGAGGCTAAAATTAGGATTCTTCAAGTAGGATTGTAGCTGACTAATGGGAGGCGTCTGATGTGGAGCCACCCGCAGTTC	EGFP fusion with NRBS-9
EGFPNRBS-131For	TTTTTCTAGATCCTGAGGCTAAAATTAGGATTCTTCAAGTAGGATTGTAGCATCGTAGGAGGAGGCAAAACATGTGGAGCCACCCGCAGTTC	EGFP fusion with NRBS-131
EGFPRev	GATATCTTACTTGTACAGCTCGTCCATGC	
GusARevLVA	AAAAGATATCTTATCAGGCTACGAGGGCGAAGGCCTGCTGGGAGGAATCGCGC	*gusA* fusion with degradation tags
GusARevAAV	TTGGTGTTGGCCTGCTTCCCGCCCTGCTGCGGAAAAGATATCTTATCAAACGGCAGCGGCGAAGGCCTGCTGGGAGGAATCGCGCTTGGTGTTGGCCTGCTTCCCGCCCTGCTGCGG	
GusARevASV	AAAAGATATCTTATCATACGGAAGCGGCGAAGGCCTGCTGGGAGGAATCGCGCTTGGTGTTGGCCTGCTTCCCGCCCTGCTGCGG	
GusASpeForw	AAAAACTAGTCGAGCAACGGAGGTAC

There are two widely used online programs (UTR Designer (https://omictools.com/utr-designer-tool) and RBS Calculator (https://www.denovodna.com/software/reverse)) that allow the translation initiation rate from a certain RBS to be predicted^1,29^. To estimate the reliability of these models, the translation initiation rate from the 70 generated RBSs was calculated using the programs mentioned above and compared to the measured GusA activity driven from these RBSs. From the data depicted in Table S1, it is clear that not all of the measured GusA expression levels corresponded to the predicted levels. For example, the N2 RBS region yielded the strongest GusA expression level, while both programs calculated the expression driven by this region to be even weaker than the control (Table S1). There was also sometimes a discrepancy in the predictions of the programs. For instance, RBS Calculator predicted that the W5 RBS should be the strongest, whereas UTR Designer predicted that the W5 RBSs should be 3 times weaker on average compared with the other strong RBSs in our library (Table S1). Considering our experimental evidence and the theoretical predictions regarding RBS activity together, we postulate that they are not correlated in all cases. The coefficient of correlation was approximately 0.32 for RBS Calculator and 0.27 for UTR Designer.

**Figure 3:**
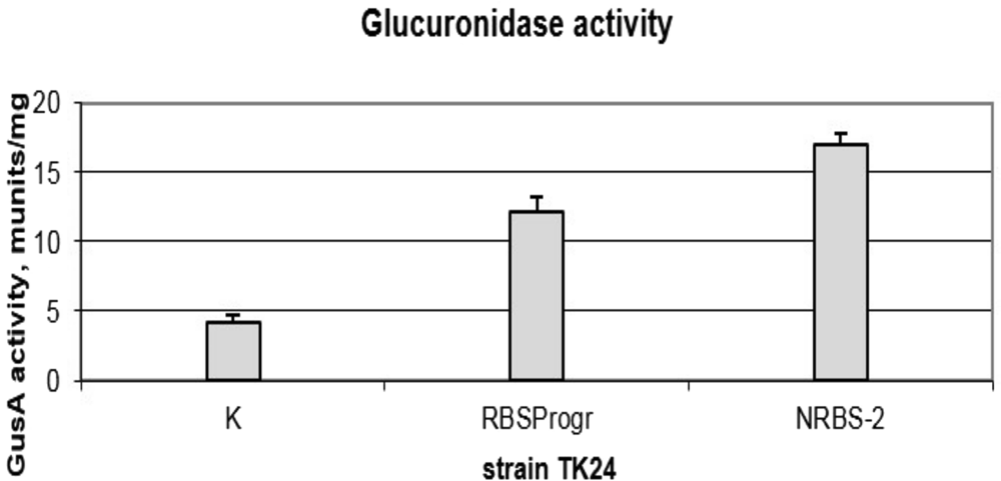
Glucuronidase activity in cell lysates of recombinant *S*. *lividans* strains containing *gusA* under the control of the P72 promoter fused to N2 (TK24 NRBS-2^+^) and programmed using RBS Calculator RBSs (TK24 RBSProgr^+^). The strains were grown in TSB medium for 2 days. Error bars indicate the standard deviations of three independent experiments.

Taking into account the ability of RBS Calculator to not only calculate but also generate an optimal RBS for a certain gene of interest, we used this program to generate an optimal RBS for the *gusA* reporter gene. This RBS was fused with the P72 weak promoter and *gusA*, and its activity was compared with the best RBS region that we succeeded in obtaining experimentally for this gene. This comparison was made based on glucuronidase activity. From the obtained data (Fig. 3) it is clear that the selected N2 RBS region was 1.5 times stronger than the programmed RBS on average. Therefore, when reliable gene expression is necessary, it is important to generate and test several RBS-surrounding regions to choose the most suitable sequence. Thus, our tool is an alternative to the programs and provides the ability to get at once plenty of RBSs with different activities for a gene of interest. Utilizing the developed tool one will get the activity of RBSs in the conditions that are required, since genetic content and the cell environment, which are not considered by *in silico* tools, can severely influence gene expression.

### The distance between the SD domain and the start codon is critical

**Figure 4:**
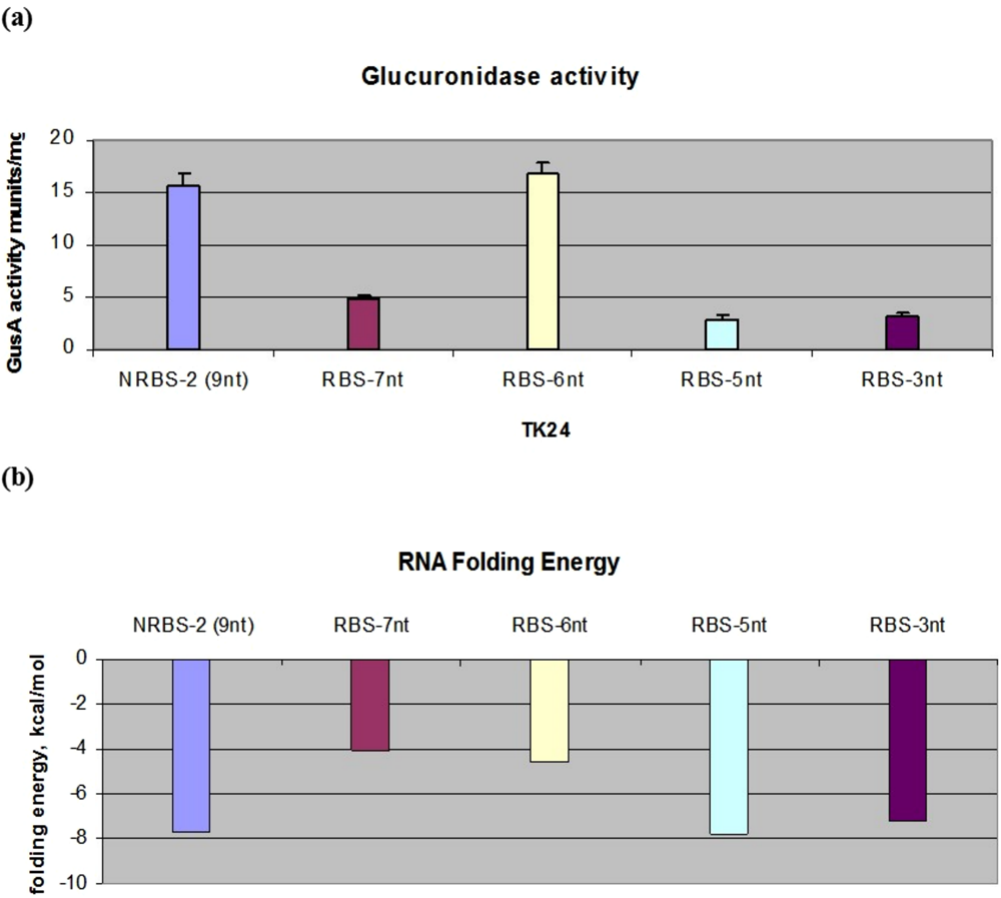
Analysis mutant RBSs that differ in the size of the insert separating the SD sequence from the ATG codon. **(a**) Glucuronidase activity in cell lysates of recombinant *S*. *lividans* strains containing *gusA* under the control of the P72 promoter fused to different mutant RBSs. The strains were grown in TSB medium for 2 days. Error bars indicate the standard deviations of three independent experiments. **(b**) RNA folding energy of mutant RBSs, calculated with NUPACK.

One of the additional parameters influencing the translation initiation rate is the distance between the SD domain and the start codon^33^. Minimal SD-AUG spacing is required, probably because the 16S rRNA and fMet-tRNA sequences must be located a certain distance apart, due to configurational limitations. Optimal spacers for *E*. *coli* are considered to range from 5 to 9 nucleotides^34^. To verify whether the same is true for *Streptomyces*, we constructed 5 RBSs that differed in the number of nucleotides located between the SD sequence and the start codon. All of these RBSs were derivatives of the strongest N2 RBS (Figs 1 and S1) that we succeeded in obtaining for *gusA*. Each of the generated RBSs was fused to the P72 promoter upstream of the *gusA* reporter gene. Subsequently, these mutant RBSs were transferred to the *S*. *lividans* TK24 strain, in which glucuronidase activity was measured (Fig. 4). Based on the measurement data (Fig. 4a), we suggest that a spacer region comprising 6 or 9 nucleotides is optimal for efficient translation in *Streptomyces*. However, we cannot rule out the influence of other factors, such as changes in mRNA stability or the mRNA folding energy. Latter is the energy required to unfold the RNA secondary structures in order to make it accessible to regulatory molecules (proteins, micro-RNAs). Less stable structure contributes to the increase of mRNA expression level^25^. We calculated the RNA folding energy for all five mutant RBSs (Fig. 4b) using NUPACK^35^ and determined that RBS-3 and RBS-5 presented among the highest RNA folding energies, similar to that of RBS2-(9 bp). However, the glucuronidase activity associated with RBS-2 was the highest and was nearly the same as that for RBS-6, which displayed an approximately 2 times lower RNA folding energy. In addition, the RNA folding energy of RBS-7 was similar to that of RBS-6, although the translation efficiency from the former was 3 times lower. These data confirmed that the observed changes in glucuronidase activity were not simply related to changes in the secondary structure of RNA but, rather, were caused by the difference in the number of nucleotides located in the spacer region. In conclusion, it is not only the RNA sequence, structure and folding energy but also the distance between the SD sequence and the start codon that plays an important role in translation initiation in *Streptomyces*.

### Development of a genetic tool – an *in vivo* RBS-selector – for the selection of an optimal RBS for any gene of interest

**Figure 5:**
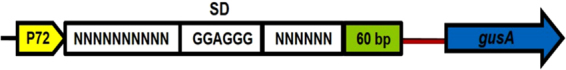
Schematic representation of the key elements of the genetic tool for the selection of RBSs for genes of interest. SD –Shine-Dalgarno domain; P72 – synthetic promoter; *gusA* – reporter gene; N – randomly synthesized nucleotides (A, T, C, G), 60 bp – first 60 bp of any gene of interest, including its start codon. The red line denotes the linker between the proximal region of the gene of interest and *gusA*.

**Figure 6:**
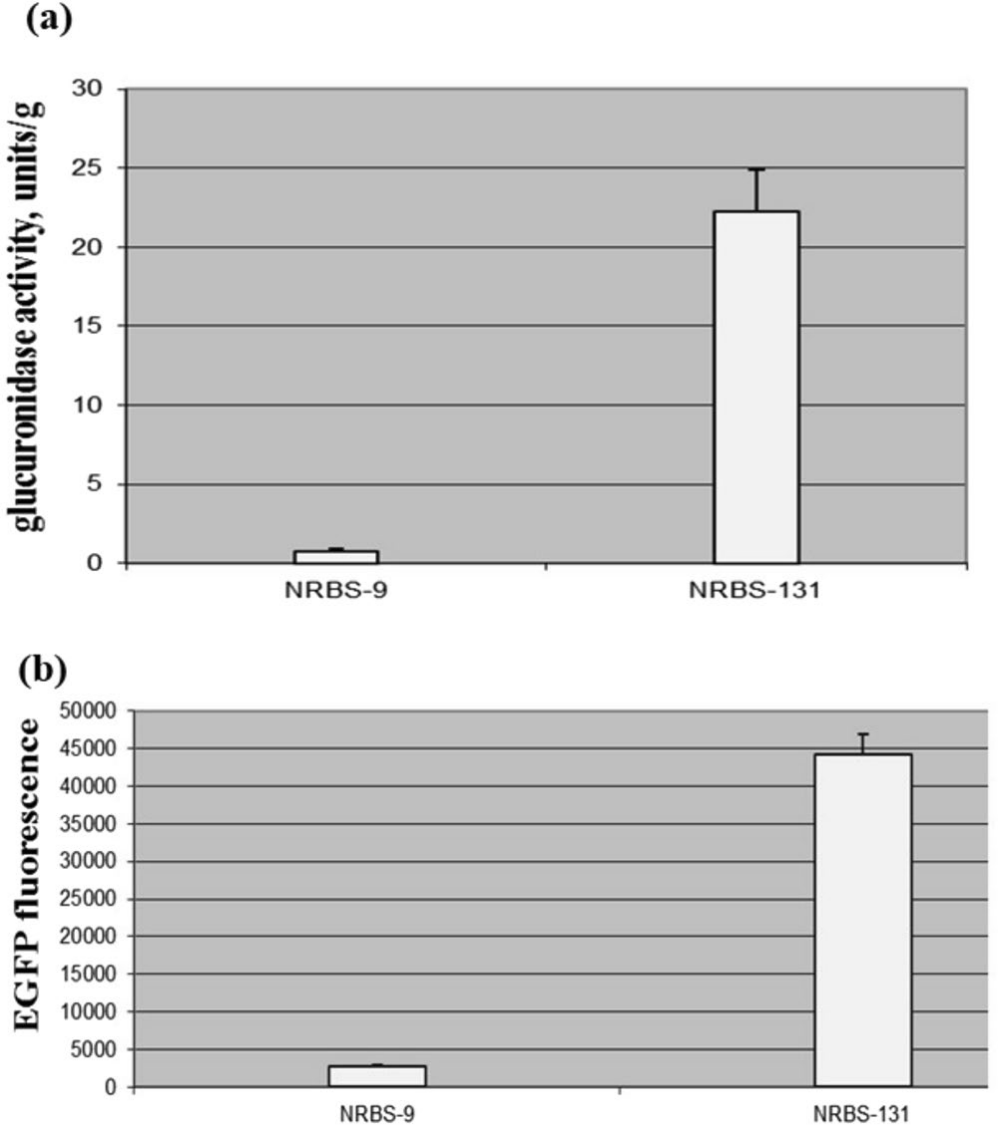
Glucuronidase activity and EGFP fluorescence in cell lysates of recombinant *S*. *albus* strains. (**a**) Glucuronidase activity in cell lysates of recombinant *S*. *albus* strains containing *gusA* under the control of the P72 promoter fused to different mutant RBSs. (**b**) EGFP fluorescence in cell lysates of recombinant *S*. *albus* strains containing *egfp* under the control of the P72 promoter fused to two different mutant RBSs. The strains were grown in TSB medium for 2 days. NRBS-9 – is the weakest RBS obtained in the study and NRBS-131 is the strongest one. Error bars indicate the standard deviations of three independent experiments.

Taking into account the various lines of evidence described above and previously showing that the 5′-UTR sequence can strongly modulate the translation initiation rate as a result of the shape and structure of mRNA and therefore plays a central role in posttranscriptional regulation, along with the fact that theoretical predictions do not always correlate with experimental data, we decided to generate a genetic tool that would allow the design and selection of an optimal RBS for any gene of interest *in vivo*. According to our predictions, this tool should reflect the influence of all major RNA components on the translation initiation rate. Through systematic analysis of the translation efficiency in *E*. *coli*, it was shown that in addition to the nucleotides surrounding the SD domain and spacer region between the SD sequence and the start codon, the first 30–60 nucleotides of the gene of interest can also strongly influence the mRNA folding energy and, consequently, ribosome access and binding to mRNA^29,36^. Therefore, an optimal RBS that is selected for one gene can be completely inefficient for another. Considering these data, the genetic tool that we designed contained the following components: P72, which is the weakest constitutive promoter and thus allows the determination of even very small changes in translation; randomly generated nucleotides surrounding the constitutive SD domain; the first 60 nucleotides, including the start codon, of any gene of interest; and the *gusA* reporter gene, to which all of these components were fused via a special linker so that the translation efficiency could be calculated indirectly (Fig. 5). Using this tool, we performed selection of an optimal RBS for *egfp* mRNA^37^. This gene was involved in the study since after the selection of the several RBSs based on glucuronidase activity it will be easy to estimate their efficiency after direct fusion with *egfp* based on fluorescence. The utilization of degenerate primers covering the first 60 nucleotides of the *egfp* gene fused with a randomly synthesized RBS and an amplified hygromycin resistance gene facilitated the rapid and easy cloning of a variety of different synthetic RBSs upstream of the *gusA* reporter gene. The obtained library of synthetic RBSs was transferred to the *Streptomyces albus* J1074 strain (Table 1) via conjugation, and the selection of an optimal RBS was performed based on glucuronidase activity (Figure S2). As a result, two RBSs (the weakest (NRBS-9) and the strongest (NRBS-131)) were chosen for further analysis (Fig. 6a). The *egfp* gene was placed under the control of the individual selected RBSs, namely NRBS-9 or NRBS-131, giving two plasmids pEGFPN-9 and pEGFPN-131 (Table 1). These plasmids were transferred into *S*. *albus* by means of conjugation. As a result, two strains *S*. *albus* pEGFPN-9^+^ and *S*. *albus* pEGFPN-131^+^ were constructed. Then relative EGFP fluorescence was measured in these strains (Fig. 6b) and compared with glucuronidase activity under the control of these RBSs. An excellent correlation between the two activities was observed. These data confirmed that we succeeded in creating a genetic tool, referred to as an *in vivo* RBS-selector, that provides the ability to perform selection of an optimal RBS for any gene of interest and enables rational control over the protein expression level. This tool requires only three steps: RBS library generation, transfer to the appropriate strain and indirect assessment of RBS activity based on the GusA assay (Figure S2).

### A library of factor-independent terminators for *S*. *lividans* TK24

**Figure 7:**
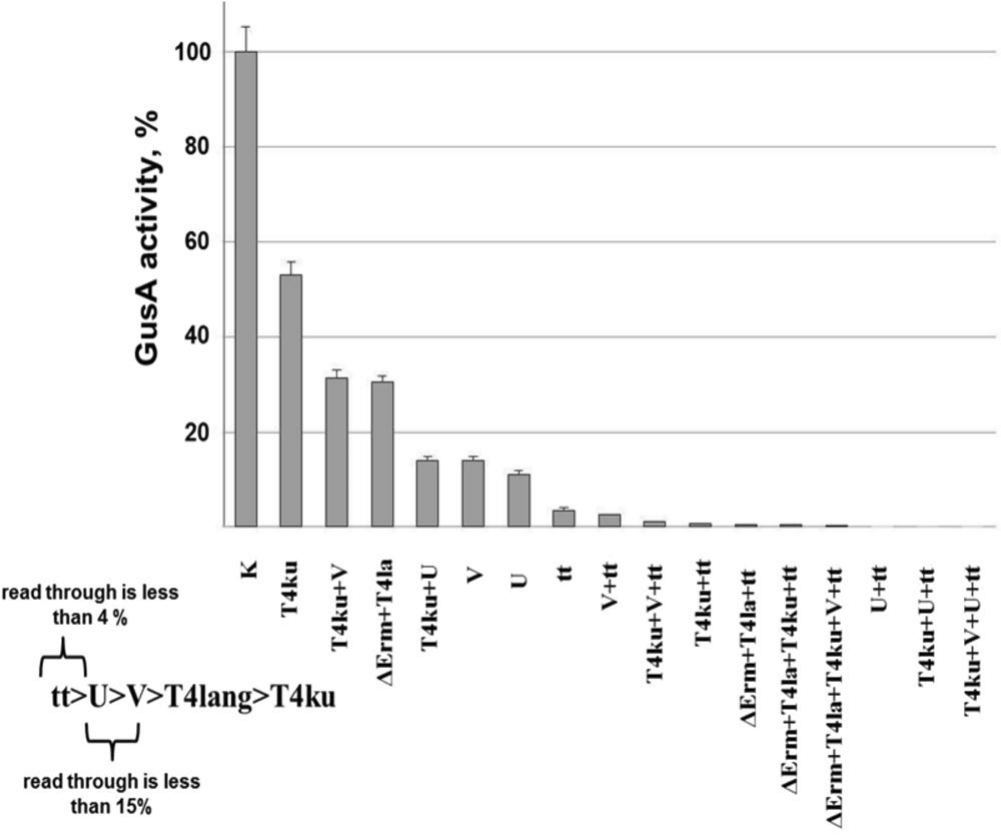
Glucuronidase activity in cell lysates of recombinant *S*. *lividans* strains containing *gusA* under the control of the P21 promoter fused to different terminators. The strains were grown in TSB medium for 2 days. Error bars indicate the standard deviation of three independent experiments. K – stands for the control strain, that expresses *gusA* under the control of the P21 promoter.

Another important type of element significantly influencing the expression of genes in prokaryotes and eukaryotes is terminators. Although the impact of terminators is relatively small compared with promoters and RBS-surrounding regions, they constitute an important component of genetic circuits. In Actinobacteria in particular, secondary metabolite gene clusters are organized into numerous operons facing each other that are controlled independently. The transcription of genes in operons is governed by different promoters and should be terminated efficiently. There are two type of terminators that function in prokaryotes: factor-dependent terminators, which rely on the special regulatory protein Rho, and factor-independent terminators, which do not require any additional protein cofactors for the termination of transcription^38–41^. The main role of terminators in operons is to terminate transcription and prevent read-through from different promoters. Because the number of sequenced genomes and, consequently, interesting gene clusters increases every year and heterologous expression of gene clusters under the control of artificial promoters is the key strategy for obtaining new metabolites, there is an urgent need for well-defined and effective terminators. There are only a few terminators that are routinely used in *Streptomyces*^42^. Thus, we decided to expand the repertoire of strong terminators for *Streptomyces* and, in particular, for *S*. *lividans*. To accomplish this aim, we chose four terminators (U, V, T4 lang and T4 kurz) from an online database (WebGeSTer Database), another terminator (tt_sbiB_) that is widely used in *Mycobacteria*^43^, and a sixth (I) that was generated in-house using an online tool. To assess the efficiency of transcription termination with the above mentioned terminators and to determine their most efficient combinations, the terminators were placed between strong constitutive promoter (P21) from our library of promoters^16^ and the *gusA* reporter gene (Figure S3). In this case, glucuronidase activity should be inversely correlated with the strength of a terminator. To test different combinations of terminators, a set of 18 plasmids was generated (Figure S3). All of the constructs were transferred to the *S*. *lividans* strain via conjugation, and the efficacy of termination was assessed based on glucuronidase activity. As a control, a plasmid carrying the *gusA* gene cloned downstream of the P21 promoter was used. The obtained results are depicted in Figs 7 and S4. GusA activity in the control strain is denoted as 100%. According to our data (Fig. 7), the tt_sbiB_ terminator was the strongest, as the resultant read-through was less than 4%. The U and V terminators were a bit weaker, with a read-through of less than 14%. The T4 lang terminator showed less efficiency, resulting in read-through in average 31%. The T4 kurz and I terminators were the weakest. In conclusion, we expanded the number of strong terminators for *S*. *lividans* and developed a genetic device that allows the assessment of terminator efficacy.

### Degradation tags for controlling gene expression at the posttranslational level

It has been shown for *E*. *coli* and some other bacteria that specific N- or C-terminal oligopeptide sequences can make stable proteins susceptible to degradation by certain intracellular, tail-specific proteases^44–46^. This feature has widely been exploited for the construction of unstable variants of reporter proteins such as an EGFP and luciferase^47–49^ in these bacteria. However, this strategy has never been used for regulating protein stability in Actinobacteria. Therefore, we decided to determine whether it is possible to use protein degradation tags to regulate gene expression at the posttranslational level and to construct unstable variants of reporter genes that might be used in the future for the investigation of temporal gene expression in Actinobacteria. For this purpose, the well-described *ssrA* degradation tag^50^ from *E*. *coli* was employed, because this type of system has also been described for Actinobacteria^51^. The *ssrA* gene encodes tm-RNA, which functions as both an mRNA and a tRNA; it also recognizes incomplete or damaged proteins and attaches a peptide tag with the sequence AANDENYALAA to the C-termini of these proteins via co-translational switching^51^. Moreover, variations in the last 3 amino acids of the peptide tag result in proteins of varying stability^48^.

**Figure 8:**
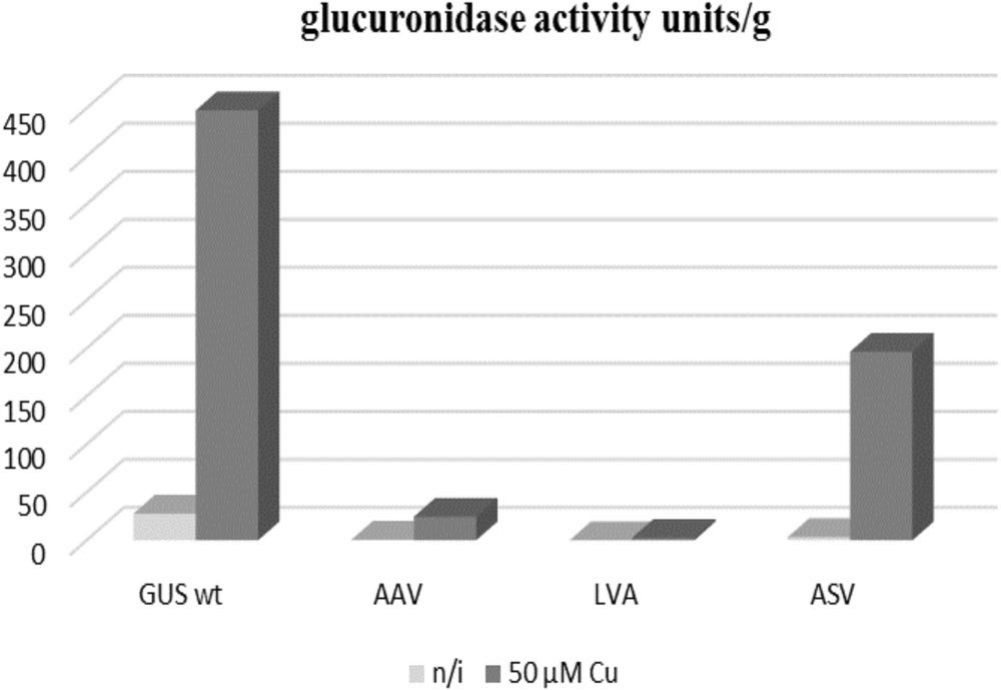
Glucuronidase activity in cell lysates of recombinant *S*. *lividans* strains containing *gusA* under the control of the inducible P21-*cmt* promoter fused to different degradation tags. GUS wt – strain that contains wild type *gusA* gene; AAV - strain that contains GusA fused with AAV C-terminal tag; LVA – strain with GusA containing LVA C-terminal tag and ASV – strain with GusA fused to ASV C-terminal tag. The strains were grown in TSB medium for 2 days in the presence or absence of the inducer.

To assess the efficiency of protein degradation in the presence of the above mentioned specific C-terminal oligopeptide extensions, these peptides were fused to GusA (Figure S5). As a result, three different plasmids, namely pGCymRP21-LVA, pGCymRP21-ASV and pGCymRP21-AAV (Table 1), were constructed. In these plasmids transcription of the *gusA* gene was under the control of the P21-*cmt* promoter^18^. The plasmids were transferred to *S*. *lividans* strains. The efficacy of degradation was assessed by measuring glucuronidase activity in the presence and absence of the inducer. As a control, a pGCymRP21 plasmid in which the *gusA* gene was cloned downstream of the P21-*cmt* promoter was employed^18^. The strains were grown in TSB medium for 48 hours in the presence or absence of the inducer (cumate). As shown by the results depicted in Fig. 8, the strain containing unmodified GusA exhibited the highest level of glucuronidase activity in the induced stage, while in the three other *S*. *lividans* strains harboring the reporter derivatives, GusA activity was 2–182-fold lower in the on (induced) state. Furthermore, *S*. *lividans* strains containing GusA with the LVA C-terminal amino acids displayed no detectable glucuronidase activity in the off stage (Fig. 8), indicating rapid degradation of the protein.

**Figure 9:**
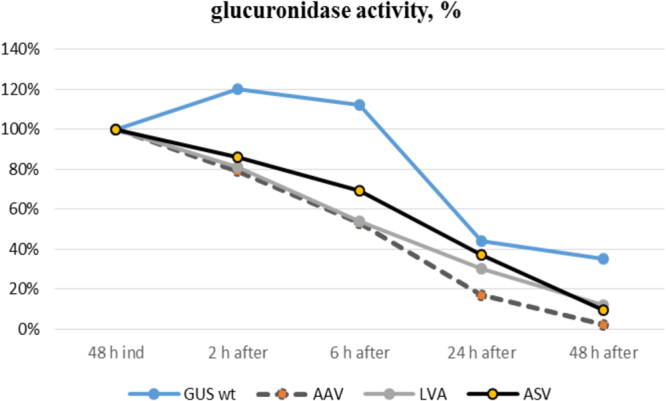
Glucuronidase activity in cell lysates of recombinant *S*. *lividans* strains containing *gusA* under the control of the inducible P21-*cmt* promoter fused to different degradation tags. GUS wt – strain that contains wild type *gusA* gene; AAV - strain that contains GusA fused with AAV C-terminal tag; LVA – strain with GusA containing LVA C-terminal tag and ASV – strain with GusA fused to ASV C-terminal tag. The strains were grown in TSB medium for 2 days in the presence of the inducer, after which the biomass was collected, washed and grown in fresh TSB medium without the inducer for 2 days.

To test the stability of the wild-type and mutant variants of GusA, we collected the biomass that accumulated after 48 hours of growth in the presence of the inducer, which was then washed twice with water, transferred it to fresh TSB medium without an inducer, and allowed to grow for 48 hours. Glucuronidase activity was measured after 2, 6, 19, 24 and 48 hours (Fig. 9). Variants of the GusA protein that contain C-terminal degradation tags were rendered unstable, while GusA with the AAV tag degraded approximately 15 times faster than the wild-type protein, and GusA-LVA and GusA-ASV degraded 2 and 3 times faster, respectively, on average. These data are in agreement with the data previously described for *E*. *coli*, *Mycobacteria* and *Pseudomonas putida*^47,48^ and again confirm the dependence of protein stability on the sequence of the amino acids in the C-terminal tag.

**Figure 10:**
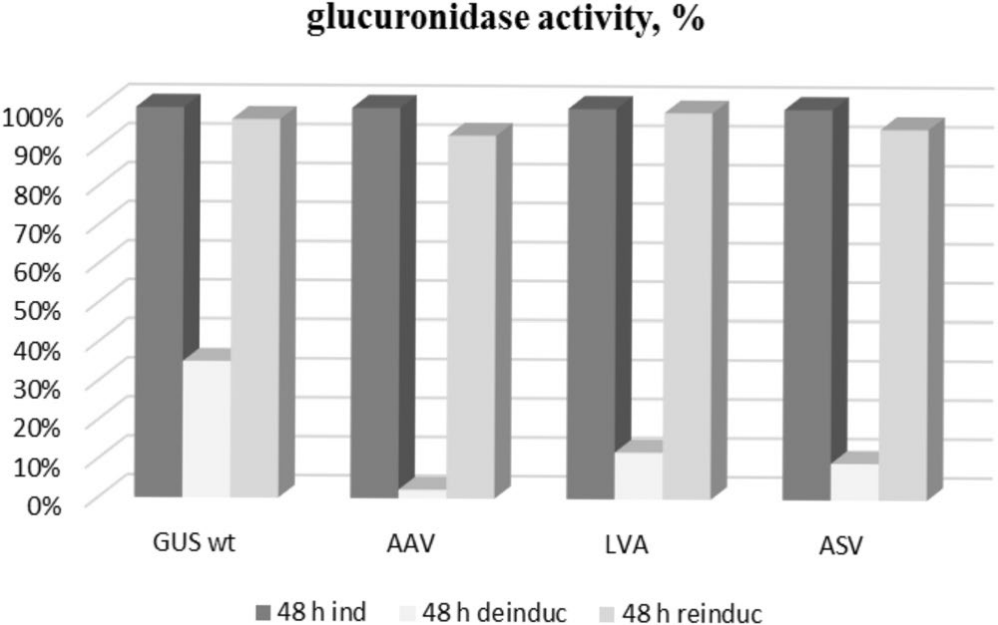
Glucuronidase activity in cell lysates of recombinant *S*. *lividans* strains containing *gusA* under the control of the inducible P21-*cmt* promoter fused to different degradation tags. GUS wt – strain that contains wild type *gusA* gene; AAV - strain that contains GusA fused with AAV C-terminal tag; LVA – strain with GusA containing LVA C-terminal tag and ASV – strain with GusA fused to ASV C-terminal tag. The glucuronidase activity of the wild type GusA in the induced stage was denoted as 100%. The strains were grown in TSB medium for 2 days in the presence of the inducer, after which the biomass was collected, washed and grown in fresh TSB medium without the inducer for 2 days; at this point, the inducer was added again, and the strains continued to grow for 2 more days.

To determine whether it was possible to restore *gusA* gene expression after GusA was degraded, we again induced *gusA* transcription with cumate for 2 days and measured GusA activity. In all strains, glucuronidase activity was restored to the maximal level observed after direct induction of the culture (Fig. 10).

In summary, we demonstrated the effectiveness of C-terminal degradation tags for the control of protein stability and used these tags to generate three rapidly degradable variants of the GusA protein, which will facilitate studies aimed at exploring dynamic changes in gene expression.

## Conclusions

Regulation of gene expression at the transcriptional level is widely performed in Actinobacteria; however, the possibility of tuning gene expression at other levels, including the posttranscriptional, translational and posttranslational, has been overlooked. In this article, we described the development of a genetic tool referred to as an *in vivo* RBS-selector that allows the strength of RBSs to be estimated and an optimal RBS with predicted activity for any gene of interest to be selected. We showed, for the first time in Actinobacteria, that RBSs can strongly influence translation efficiency, decreasing it to 0 in certain cases, and that this type of controlling element might be used for tuning gene expression at the translational level. Furthermore, we demonstrated that there is only a moderate correlation between the strength of an RBS observed in experiments and its activity predicted using online tools, such as UTR Designer and RBS Calculator, which is in accord with the recently reported data from *Streptomyces coelicolor*^52^. In addition, an RBS selected for *egfp* based on glucuronidase activity was shown to be approximately 1.5 times stronger than the best RBS designed using RBS Calculator, indicating that genetic content and the cell environment can influence gene expression, which are not considered by *in silico* tools. We also constructed a library of RBSs for the *gusA* gene and, consequently, obtained numerous reporters with different sensitivities.

In addition, a genetic tool for the reliable estimation of terminator strength was developed and used for the assessment of termination efficiency in *S*. *lividans*. We employed this tool to characterize several well-defined strong terminators that cause 2–26-fold reductions of gene expression and are characterized by sufficient sequence diversity to reduce homologous recombination when used together with a synthetic operon. For the first time, the *ssrA* degradation tags were employed for the regulation of protein stability and, thus, control of gene expression at the posttranslational level in Actinobacteria. Three new variants of the unstable GusA protein were generated, which will facilitate dynamic monitoring of changes in gene transcription. The described features make the above-described tools very promising for metabolic engineering and biotechnology of *S*. *lividans* TK24 and other Actinobacteria.

## Materials and Methods

### Bacterial strains and growth conditions

The bacterial strains used in this study are listed in Table 1. *E*. *coli* strains were grown in Luria–Bertani (LB) broth medium. When required, antibiotics (Sigma, USA; Roth, Germany) were added to cultures at the following concentrations: 100 μg ml^−1^ ampicillin, 50 μg ml^α^ kanamycin, 50 or 120 μg ml^−1^ hygromycin, 50 μg ml^−1^ apramycin.

For conjugation, *Streptomyces albus*, *Streptomyces lividans* strains were grown on oatmeal or mannitol soy (MS) agar^42^ for sporulation. For glucuronidase activity measurement strains were grown in liquid tryptic soy broth (TSB).

### Recombinant DNA techniques

Chromosomal DNA from *Streptomyces* strains and plasmid DNA from *E*. *coli* were isolated using standard protocols^42,53^. Restriction enzymes and molecular biology reagents were used according to the recommendations of the supplier (Thermo Fisher Scientific, Germany, NEB, England).

### Generation and cloning of the synthetic RBSs for the *gusA* reporter gene

A version of the *gusA* gene without promoter and RBS was synthesized using primers XbagusAForw and EVgusARev (Table 2). Obtained 1.8 kb fragment was digested with *Xba*I and *Eco*RV sites and ligated into respective sites of pGUS vector^24^. As a result, a pGUSbezRBS (Table 1) plasmid was constructed.

The library of synthetic RBSs was constructed using two types of degenerate primers, in which N is any of the four base pairs (A, T, G, C) and W – only adenine or thymine (A or T). Using primer pairs XbaRBSForw and NdeNRBSRev or XbaRBSForw and NdeWRBSRev (Table 2), respectively, the hygromycin resistance gene fused with the P72 weak promoter^16^ was amplified by PCR, digested with *Nde*I and *Xba*I and cloned into the respective sites of pGUSbezRBS vector, yielding two types of plasmids: pGUSNRBS and pGUSWRBS (Fig. 1a). Hygromycin gene fused only to the P72 promoter without RBS was amplified using primer pair XbaRBSForw and NdeP72bezRBS (Table 2). The obtained fragment was cut with *Xba*I/*Nde*I and cloned into the respective sites of pGUSbezRBS. As a result, plasmid pGUSP72bezRBS that contained *gusA* gene under the control of the P72 promoter, however without RBS, was constructed (Table 1). Plasmids pGUSP72bezRBS, pGUSNRBS or pGUSWRBS were introduced into the *S*. *lividans* TK24 strain via tri-parental conjugation. In this type of mating *E*. *coli* strain ET12567 x pUB307 was used as a helper to transfer the plasmids from *E*. *coli* strain DH5α into *S*. *lividans*.

To directly compare the activity of the synthetic RBSs designed for the *gusA* gene using the RBS calculator online tool (https://www.denovodna.com/software/reverse) with the RBSs we obtained on practice, former were fused with the P72 promoter and hygromycin resistance gene, and cloned into the pGUSbezRBS vector using the same algorithm. The forward primer XbaRBSForw in this case was always the same and the reverse primer included the sequence of *in silico* generated RBSs. In this way, Prog1 RBS was amplified using primers XbaRBSForw and RBSProg1Rev (Table 2). The obtained 1.3 kb fragment was digested with *Nde*I and *Xba*I and cloned into the respective sites of pGUSbezRBS, yielding pGUSRBSProg1 (Table 1).

### Generation and cloning of synthetic RBSs with different spacing between the Shine-Dalgarno (SD) sequence and the start codon of the *gusA* reporter gene

To construct a series of plasmids that differ in the size of the insert separating the SD from the ATG, the strongest RBS, NRBS2, was used. For this purpose, the forward primer XbaRBSForw was always the same, and the reverse primer included the sequences of generated RBSs differing in the length of spacing. To synthesize an RBS with 8-bp spacing, the reverse primer NdeRBS8Nrev was used; for 7-bp spacing, NdeRBS7Nrev was used; for 6-bp spacing, NdeRBS6Nrev; for 5-bp spacing, NdeRBS5Nrev; and for 3-bp spacing, NdeRBS3Nrev (Table 2). The amplified fragments obtained using pairs of the abovementioned forward and reverse primers were digested with *Nde*I/*Xba*I and cloned into the respective sites of pGUSbezRBS. Consequently, the plasmids pGUSNRBS-8 bp, pGUSNRBS-7 bp, pGUSNRBS-6 bp, pGUSNRBS-5 bp, and pGUSNRBS-3 bp (Table 1) were constructed and used in further experiments.

### Generation, cloning and selection of synthetic RBSs for the *egfp* gene based on the activity of *gusA*

To obtain a fusion of the ampicillin resistance marker with the P82 synthetic promoter^16^, the primer pair ApForw and ApP82Rev (Table 2) was used. The amplified 1.3-kb fragment was cloned into the *Eco*RV site of the pSET152 integrative vector, yielding pSETP82Ap (Table 1).

A library of synthetic RBSs was constructed using degenerate primers, in which N is any of the four base pairs. Using the primers ApForw and EGFPNRBSRev, the ampicillin resistance gene fused with the P82 weak promoter^16^ and randomly generated RBSs was amplified via PCR, digested with *Eco*RV and *Xba*I and cloned into the respective sites of the pGUSHL4aadA vector^24^, yielding the pGUSNRBSEGFP plasmid (Table 1, Fig. 5). The pGUSNRBSEGFP plasmid was introduced into the *S*. *albus* strain via tri-parental conjugation.

Two synthetic RBSs (the strongest and the weakest) were selected based on *gusA* activity and used in further experiments. To fuse the *egfp* gene with these RBSs and the P82 promoter, two primer pairs were used: EGFPRev and EGFPNRBS-9For for the weakest RBS and EGFPRev and EGFPNRBS-131For for the strongest RBS. The obtained amplified fragments were digested with *Xba*I and cloned into an *Xba*I/*Eco*RV-hydrolyzed pGUS vector, yielding pEGFPN-9 and pEGFPN-131 (Table 1).

### Construction of unstable variants of the GusA reporter

To translationally fuse the *gusA* gene with C-terminal degradation tags, the following reverse primers were used: GusARevLVA, GusARevAAV, and GusARevASV (Table 2). The forward primer was the same in all cases – GusASpeForw (Table 2). As a result of PCR amplification, three different 1.9-kb fragments were obtained, which were then digested with *Spe*I/*Eco*RV and cloned into the respective sites of the pGCymRP21 plasmid^18^, yielding plasmids pGCymRP21-LVA, pGCymRP21-ASV, and pGCymRP21-AAV (Table 1).

### Calculation of terminator efficiency

In our reporter construct, terminators are placed between the strongest synthetic promoter (P21)^16^ and the *gusA* reporter gene. As a control, a plasmid containing the P21 promoter fused to the *gusA* gene, without terminators between them, was used. Presence of terminators between the P21 promoter and the *gusA* gene will reduce the fraction of *gusA* transcripts, preventing read-through and consequently reducing glucuronidase activity. Therefore, the ratio of glucuronidase activity in the presence of a terminator (GUSTerminator) to *gusA* activity in the control plasmid (GUSControl) was used for the calculation of terminator read-through (TR), which is reported as % values. Based on these values, terminator efficiency was estimated as follows:

TR (%) = GUSTerminator/GUSControl × 100

### Assessment of RBS and terminator strength (GUS assay)

For direct detection of glucuronidase activity, 1- to 5-day plates were flooded with 5-bromo-4-chloro-3-indolyl glucuronide (X-Gluc) solution and incubated at 28 °C for 1–4 h. A 1 M X-Gluc stock solution was prepared in dimethyl sulfoxide. The final concentration of the X-Gluc solution used for flooding plates was 20 or 200 mM.

For the quantitative measurement of GusA activity, 1 ml of 24-h seed cultures of the *S*. *lividans* TK24 or *S*. *albus* J100 recombinant strains was inoculated into 25 ml of TSB. The cells were grown for 1 or 2 days. A 5-ml aliquot of the culture was harvested via centrifugation (6,000 × *g* for 10 min) and used for the measurement of glucuronidase activity, as described in Horbal *et al*., 2014. In case of all samples 2 ml of the culture broth was centrifuged, the supernatant was discarded and the biomass was dried for 2 days at 75 °C. All measurements were normalized to dry weight, and the presented results are from three independent experiments. Microsoft Excel was employed for statistical analysis.

## Electronic supplementary material

Supplementary Material
