# Supplementary material for: A set of synthetic versatile genetic control elements for the efficient expression of genes in Actinobacteria
Source: Sci Rep. 2018 Jan 11;8:491. doi: 10.1038/s41598-017-18846-1 (PMC5765039; doi:10.1038/s41598-017-18846-1)
Supplement: Supplementary file 1 — Supplementary Material [file 41598_2017_18846_MOESM1_ESM.pdf]

Supplementary Materials

A set of synthetic versatile genetic control elements for the efficient expression of genes in Actinobacteria

Lilya Horbal[1], Theresa Siegl[1], Andriy Luzhetskyy[1,2*]

[1]Department Microbial Natural Products, Actinobacteria Metabolic Engineering Group, Saarland University,
[2]Helmholtz-Institute for Pharmaceutical Research Saarland (HIPS) Helmholtz Center for Infectious Research (HZI), Campus C2.3, 66123, Saarbrücken, Germany

*Corresponding author – Prof. Dr. Andriy Luzhetskyy

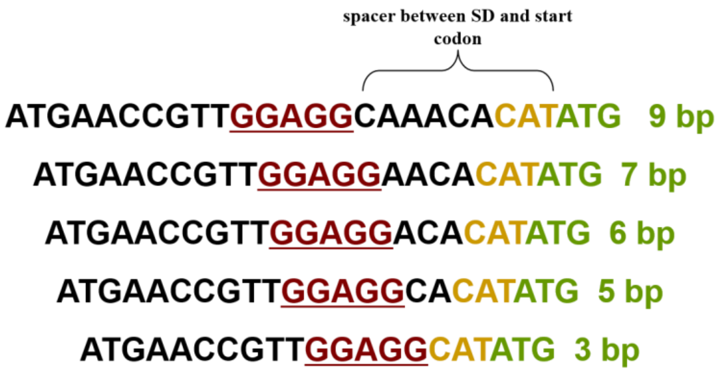

Figure S1. The sequences of the RBSs that differ in the length of spacer between the SD domain and the start codon.

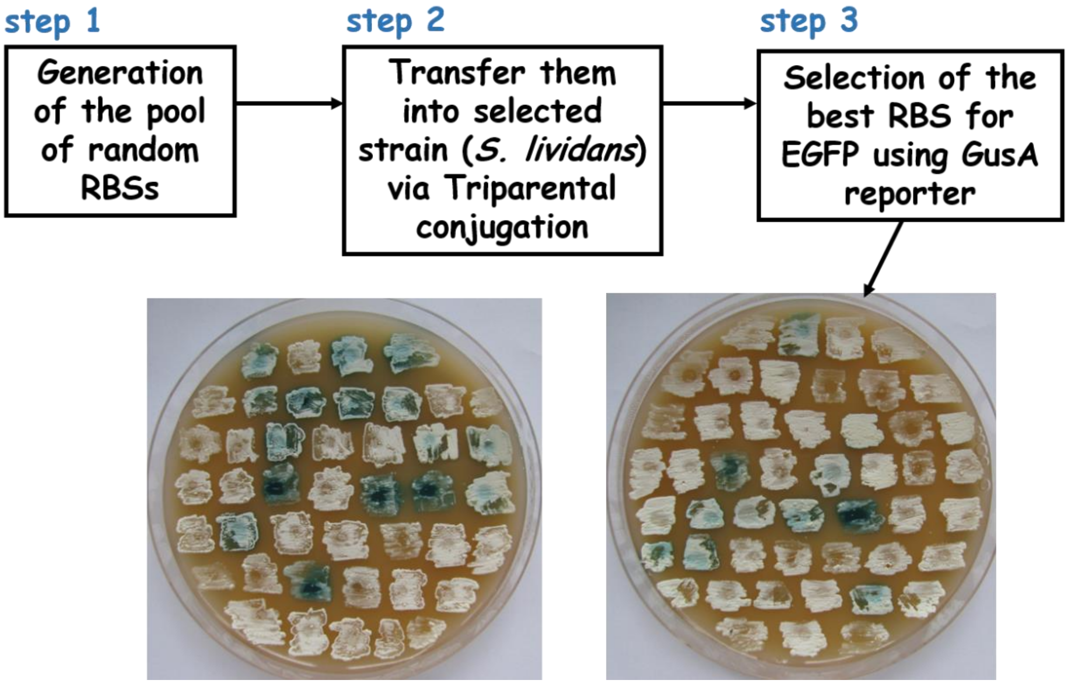

Figure S2. Key steps on the way to optimal RBS for gene of interest.

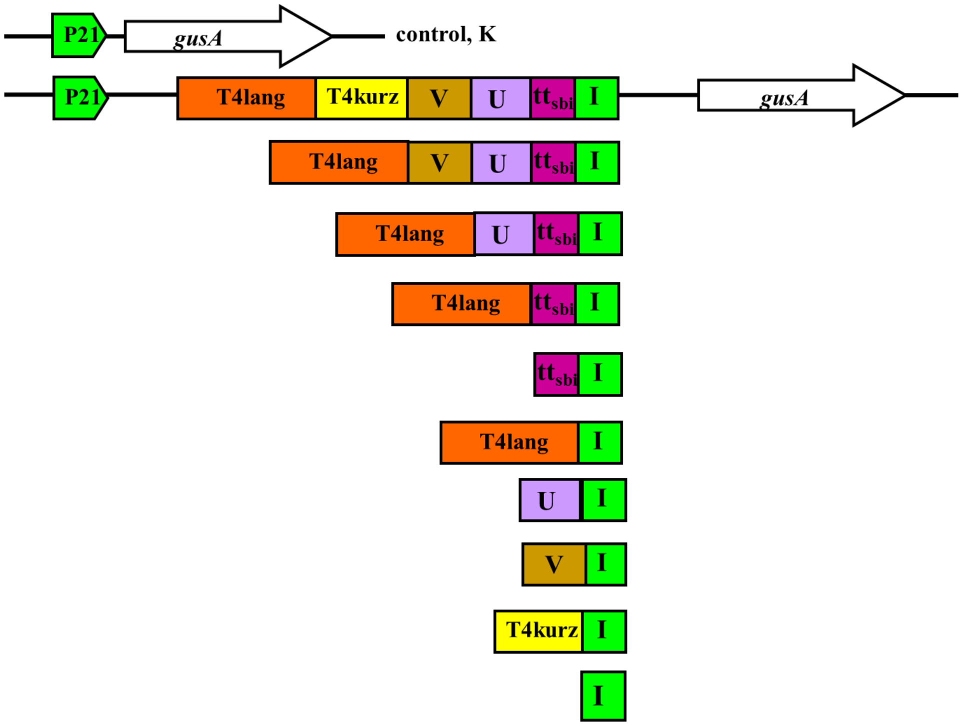

Figure S3. Schematic representation of the constructs for the analysis of termination efficiency of transcription: P21, strong synthetic promoter; *gusA*, reporter gene; terminators – T4 lang, T4 kurz, V, U, tt_{sbi}, I.

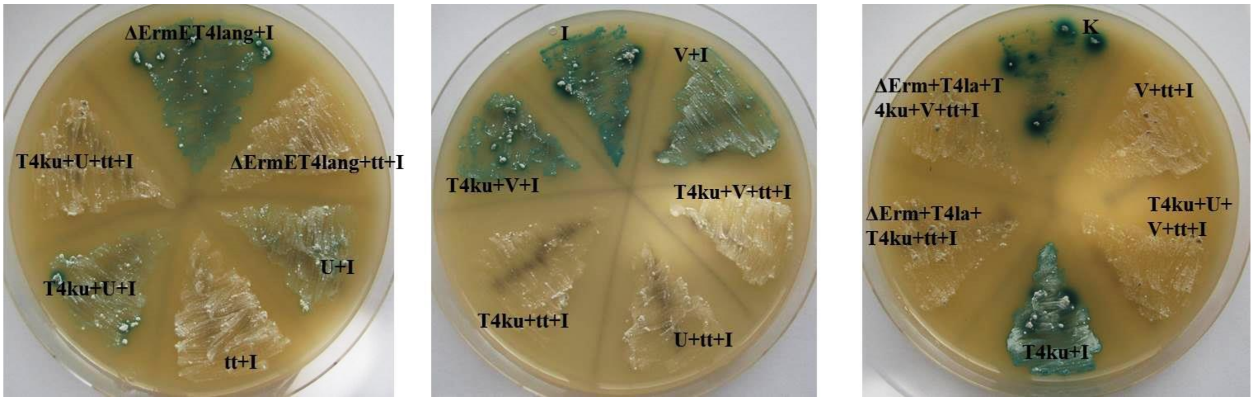

Figure S4. *S. lividans* strains containing different combinations of terminators grown for 2 days on MS medium with X-Gluc.

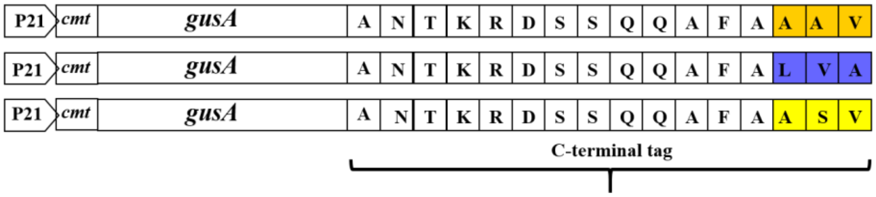

Figure S5. Schematic representation of the constructs containing the *gusA* gene fused with different degradation tags.

Table S1. RBS efficiency based on the measurement of GusA activity or theoretical predictions of translation initiation rates using RBS Calculator or UTR Designer.

*the activity of the control RBS was denoted as 100 %

RBS	Glucuronidase activity, units/g	Normalized to the activity of the control RBS, %	Translation Initiation Rate (RBS calculator / UTR designer)	Sequence 5'-3'
**No RBS**	0	0		
**N4**	0	0	254.47 / 34538	ACTAGGTGGATGGAGGTAGTCACATATG
**N20**	0	0	232.56 / 34538	AGACCCACTCTGGAGGATAGATCATATG
**N37**	0	0	266.18 / 7418	ACTAAACCCCGGGAGGCCAACGCATATG
**N26**	0.99	12.86	4.24 / 4742	ATCAGAGCTGTGGAGGCCACTCCATATG
**N17**	1.2	15.58	60.28 / 11285	ATGGCCAGCCGGGAGGGCCCCCCATATG
**N45**	1.44	18.7	65.96 / 8296	ACGTCGCATACGGAGGCGTTTTCATATG
**N23**	1.8	23.37	19.15 / 7418	ACACATCCATCGGAGGGTTTGGCATATG
**N46**	1.8	23.37	148.28 / 16232	ACAATAAGCCCGGAGGCCGATACATATG
**N1**	1.88	24.41	123.85 / 20302	ACCCAAGAGATGGAGGGCCCCCCATATG
**W9**	1.96	25.45	8139.74 / 990181	AAAATAAAAAAGGAGGAAAAATCATATG
**N6**	1.96	25.45	14.94 / 42007	AGACCTTTGTTGGAGGTCCACTCATATG
**N27**	2.2	28.57	1286.05 / 156365	ACCAACAGGTAGGAGGTAAACCCATATG
**W56**	2.84	36.88	254.47 / 212685	ATTTTTTAATTGGAGGTTTTTTCATATG
**W2**	3.48	45.19	2206.99 / 382633	ATTATTAAATAGGAGGAATATTCATATG
**N34**	3.72	48.31	897.21 / 57137	ACAACGTCGGAGGAGGATACGACATATG
**W34**	4.23	54.93	522.82 / 218716	ATATTTTTTTAGGAGGATATAACATATG
**W27**	4.24	55.06	1762.28 / 289289	AATAAAATAATGGAGGAAAAAACATATG
**W39**	4.38	56.88	278.43 / 26113	ATTAATTTTTTGAGGTATTTACATATG
**N3**	4.51	58.57	203.19 / 152053	ACACCCCACCAGGAGGATCTTCCATATG
**W15**	4.68	64.68	16.34 / 195570	ATTTTTTTTTTGGAGGATTTTTCATATG
**N19**	5.28	68.57	232.56 / 14514	AAAGAGCCAAGGAGGGCGGCCCATATG
**N35**	5.75	74.68	194.25 / 30883	ATTCGCCGTTTGGAGGACGCCCCATATG
**W3**	6.04	78.44	1471.95 / 160800	ATTATTTTTTTGGAGGTTTTATCATATG
**W28**	6.15	79.87	36.74 / 212685	AATTTTTTTTTGGAGGTTTTTTCATATG
**W18**	6.21	80.65	5428.8 / 707904	AAAAAAAATAAGGAGGAAAAAACATATG
**W53**	6.3	81.82	36.74 / 212685	AATTTTTTTTTGGAGGTTTTTTCATATG
**W51**	6.31	81.95	436.69 / 160800	ATTTATTTTTTGGAGGTTATTTCATATG
**W59**	6.31	81.95	572.06 / 212685	ATTTATTTTTTGGAGGTTTTTTCATATG
**W61**	6.54	84.94	3620.74 / 427921	AATAAAAAATAGGAGGAATAATCATATG
**W65**	6.59	85.58	1762.28 / 218716	AAAATATTAATGGAGGAATAATCATATG
**W66**	7.1	92.21	1762.28 / 289289	ATAAAAAAAATGGAGGAAAAAACATATG
**N47**	7.46	96.88	90.38 / 19742	AGCAGCCGATAGGAGGGACCTTCATATG
**W58**	7.19	93.38	8139.74 / 990181	AATAAAAAAAAGGAGGAAAAAACATATG
**W36**	8	104	6798.76 / 791688	AAAATTATTAAGGAGGTAAAAACATATG
**W69**	8.17	106.1	897.21 / 206819	AAAATTTATTTGGAGGTATTTACATATG
**W37**	8.42	109.4	2333.71 / 990181	AAAAAAAAAAGGAGGAAAAATCATAAG
**W21**	8.59	111.6	8139.74 / 990181	AAAAAAATAAAGGAGGAAAATACATATG
**W62**	8.78	114.02	5428.8 / 707904	AAAAATATTAAGGAGGAAAAAACATATG

**W22**	9.12	118.4	5428.8 / 707904	AAAAAAAAAATAAGGAGGAAAAAACATATG
**W26**	9.4	122.07	5940.11 / 565996	AAAAAAAAAAAGGAGGATAAAACATATG
**N29**	9.47	122.99	1175.35 / 79921	AGTGAAAAAAAGGAGGGACGCCCATATG
**N51**	9.69	125.84	272.48 / 8077	AAACGGAAATAGGAGGAGGCAGCATATG
**W25**	9.74	126.49	8139.74 / 990181	ATAAAAAAAAAGGAGGAAAAAACATATG
**W17**	10.48	136.1	8139.74 / 20878	AAAAAAAAAAAGGAGGAATATACATATG
**W23**	10.95	142.21	8139.74 / 20878	AAAAAAAAAAAGGAGGAAAATACATATG
**N13**	11.23	145.84	399.1 / 2327	ACGTATTCTAGGGAGGCCATGCCATATG
**N12**	11.29	146.6	120.92 / 1606	AACGTACGGACGGAGGAGAGTCCATATG
**W12**	11.6	151	171.56 / 33101	ATTTTTTTTTTGGAGGGTTTTTTCATATG
**W24**	11.68	151.69	8139.74 / 20878	AAAAAAAAAAAGGAGGAAAAAACATATG
**W52**	12	155.84	1843.4 / 24038	ATTTATTTTTTTGGAGGATAATACATATG
**W4**	13.86	180	8139.74 / 20878	AAAAAAAAAAAGGAGGAAATAACATATG
**W20**	14.24	184.9	8139.74 / 20878	AAAAAAAAAAAGGAGGAAAATACATATG
**W5**	16.14	209.6	8139.74 / 289289	ATAAAAAAAATGGAGGAAAAAACATATG
**N16**	20.53	266.6	13.36 / 14514	GAGATTCTTTAGGATGAGAAACCATATG
**N2**	24	311.69	522.82 / 20878	AATGAACCGTTGGAGGCAAACACATATG
